# Priorities and opportunities for palliative and end of life care in United Kingdom health policies: a national documentary analysis

**DOI:** 10.1186/s12904-021-00802-6

**Published:** 2021-07-14

**Authors:** Katherine E. Sleeman, Anna Timms, Juliet Gillam, Janet E. Anderson, Richard Harding, Elizabeth L. Sampson, Catherine J. Evans

**Affiliations:** 1 Faculty of Nursing, Midwifery and Palliative Care, King’s College London, Cicely Saunders Institute, Bessemer Road, London, SE5 9PJ UK; 2 Florence Nightingale Faculty of Nursing, Midwifery and Palliative Care, James Clerk Maxwell Building, 57 Waterloo Road, London, SE1 8WA UK; 3 School of Health Sciences, City, University of London, Northampton Square, London, EC1V 0HB UK; 4 Marie Curie Palliative Care Research Department, Division of Psychiatry, University College London, London, UK; 5 Barnet Enfield and Haringey Mental Health Trust Liaison Psychiatry Team, North Middlesex University Hospital, London, UK; 6 Sussex Community NHS Foundation Trust, Brighton, UK

**Keywords:** Palliative, Health policy, End of life care, Documentary analysis

## Abstract

**Background:**

Access to high-quality palliative care is inadequate for most people living and dying with serious illness. Policies aimed at optimising delivery of palliative and end of life care are an important mechanism to improve quality of care for the dying. The extent to which palliative care is included in national health policies is unknown. We aimed to identify priorities and opportunities for palliative and end of life care in national health policies in the UK.

**Methods:**

Documentary analysis consisting of 1) summative content analysis to describe the extent to which palliative and end of life care is referred to and/or prioritised in national health and social care policies, and 2) thematic analysis to explore health policy priorities that are opportunities to widen access to palliative and end of life care for people with serious illness. Relevant national policy documents were identified through web searches of key government and other organisations, and through expert consultation. Documents included were UK-wide or devolved (i.e. England, Scotland, Northern Ireland, Wales), health and social care government strategies published from 2010 onwards.

**Results:**

Fifteen policy documents were included in the final analysis. Twelve referred to palliative or end of life care, but details about what should improve, or mechanisms to achieve this, were sparse. Policy priorities that are opportunities to widen palliative and end of life care access comprised three inter-related themes: (1) integrated care – conceptualised as reorganisation of services as a way to enable improvement; (2) personalised care – conceptualised as allowing people to shape and manage their own care; and (3) support for unpaid carers – conceptualised as enabling unpaid carers to live a more independent lifestyle and balance caring with their own needs.

**Conclusions:**

Although information on palliative and end of life care in UK health and social care policies was sparse, improving palliative care may provide an evidence-based approach to achieve the stated policy priorities of integrated care, personalised care, and support for unpaid carers. Aligning existing evidence of the benefits of palliative care with the three priorities identified may be an effective mechanism to both strengthen policy and improve care for people who are dying.

**Supplementary Information:**

The online version contains supplementary material available at 10.1186/s12904-021-00802-6.

## Introduction

Palliative and end of life care is a neglected global health issue [1]. Worldwide approximately 25 million people die each year with serious health related suffering, defined as suffering resulting from illness or injury which cannot be alleviated without medical intervention [2]. This number is projected to double by 2060 [3]. While palliative care is increasingly recognised as a human right [4], it is estimated that just 14% of the people who need palliative care globally receive it [5].

Palliative care is the holistic care of people living with life-threatening illness. The focus of palliative care is to improve quality of life, and relieve suffering, for both patients and their carers. Palliative care may be applicable throughout the course of a patient’s illness, but needs increase towards the end of life [6]. Growing evidence supports the effectiveness and cost-effectiveness of specialist palliative care. Patients receiving early specialist palliative care show improvement in a range of outcomes including physical symptom control [7, 8], survival [8–10], and quality of life [7, 9, 11], while carers express increased satisfaction [7] and decreased depression [10]. Palliative care is also likely to be cost-effective for society [12]. Consequently, there have been high profile inter-governmental calls to strengthen palliative care provision [13] and palliative care is now integrated into the World Health Organisation’s concept of Universal Health Coverage [14].

Healthcare policies are defined as ‘decisions, plans, and actions that are undertaken to achieve specific health care goals within a society’ [15]. Policies promote improvement in health and care through advancement and implementation of laws, regulations and practices that influence development of systems, as well as organisational and behaviour change [16]. Health policy frequently uses explicit targets, in terms of access and outcomes, to achieve goals [17]. Thus, policy is regarded as an essential tool to enact positive reform. While there is clarity on the desired qualities of policy making (that it should be outward-looking, evidence-based, inclusive, forward-looking, innovative and joined up [18]), it is less clear how to achieve these. Policies aimed at improving access to palliative and end of life care are considered an important mechanism to ensure quality of care for the dying [19]. Documentary analysis is increasingly used to study policy making, and can provide insights relating to agenda-setting [20], policy priorities [21], policy and evidence gaps [22], evolution of policy discourse [23], and mechanisms of and opportunities for knowledge transfer.

The United Kingdom (UK) has been described, using an international ranking exercise based on the healthcare environment, human resources, community engagement, and affordability and quality of care, as providing the best ‘quality of death’ in the world [19]. In the UK, specialists in palliative care work across acute hospital, community and inpatient hospice settings, providing direct care to patients as well as indirect care through education and training of non-specialists. Around 30% of hospice funding is from the government, with 70% from charitable sources [24]. It is estimated that around 75% of people in England and Wales would benefit from palliative care as they approach the end of life, and palliative care needs are projected to increase 25% by 2040 with the greatest projected increase among people with dementia [25]. A previous documentary analysis showed that palliative and end of life care are rarely prioritised in the 150 regional Health and Wellbeing Board policies in England [26]. Whether and how palliative care is a priority in the UK or its devolved nations’ (England, Scotland, Northern Ireland, Wales) health policies is not known. Furthermore, understanding how palliative care aligns with wider health priorities may identify opportunities to deliver policy support for palliative and end of life care at a national level.

The aim of this study was to undertake a documentary analysis of UK and its devolved nations’ health policies to identify priorities and opportunities for palliative and end of life care. The objectives were 1) to describe the extent to which palliative and end of life care is referred to in national health policies, and 2) to explore policy priorities that could be used as opportunities for widening access to palliative and end of life care. Questions guiding the analysis were: what specific aspects of palliative and end of life care are referred to in national policy documents? In what context is palliative and end of life care explicitly mentioned? How does palliative and end of life care align with current policy priorities? What are the policy priorities that could be used as vehicles to widen access to palliative and end of life care?

## Methods

### Design

Documentary analysis consisting of 1) summative content analysis to describe the extent to which palliative and end of life care is referred to in national health policies, and 2) thematic analysis to explore policy priorities that could be used as opportunities for widening access to palliative and end of life care.

### Data acquisition

Potentially relevant policy documents were identified through: (i) targeted website searches (e.g. relevant government bodies and ministries); (ii) Google search to identify documents missed in the first step; and (iii) expert consultation with researchers and clinical academics in palliative care and policy makers. This search strategy reflects that recommended to identify web-based resources in grey literature [27]. The resulting ‘long list’ of policy documents was discussed by team members (AT, KES, CJE) to determine the eligible documents for the final analysis.

### Inclusion/exclusion criteria

Documents were included if they were (i) government strategies published from 2010 onwards and available at the time of the search (July 2019), (ii) nationally relevant (i.e. covering either the whole of the UK, or the devolved nations), and (iii) health and/or social care strategies. We focused on strategies published from 2010 as we were interested in a relatively contemporary understanding of policy. Our intention was to gain a broad overview of content relating to, and opportunities for, palliative and end of life care using a sample of the most relevant documents, rather than an exhaustive search. We excluded strategies that focused on specific patient groups (e.g. older people) or those with a disease-specific focus such as dementia or cancer strategies as our intention was to gain a broad population overview of policy initiatives rather than condition or population specific detail. We excluded those that focused specifically on palliative or end of life care as we were interested in understanding how palliative care aligns with broader health and social care priorities, and identifying health priorities that could be used as drivers to widen access to palliative and end of life care.

### Data extraction and analysis

Data extraction and analysis was in two phases, in line with the objectives. Data was stored and coded in Excel (Microsoft, USA).

The first phase examined the extent to which palliative and end of life care is referred to in policies. For this phase summative content analysis was used to quantify the frequency of key terms relating to palliative and end of life care, and to identify the context of these terms. Summative content analysis is particularly suited to documentary analysis [26, 28]. It starts with identification of the frequency with which specific words or phrases are used, and this then forms the basis for more in-depth qualitative analysis exploring the context within which these words are used [29]. For this phase, the documents were systematically searched electronically using key terms to identify relevant content. The key terms were palliat*, end of*, terminal, hospice, bereave*, death, die, and dying, based on those used in a previous study [26]. A data extraction form was used to record information on the document title, date of publication, authors, jurisdiction (i.e. UK wide, single nation), intention of the policy, and the number of times the key words were mentioned in a relevant context (we ignored mentions of key words in non-relevant contexts, e.g. ‘accidental deaths’). To facilitate analysis of the context within which key terms were mentioned, where these were identified the relevant section of the policy was extracted to produce a resource folder from which data familiarisation and qualitative analysis occurred. We were particularly interested in understanding if palliative and end of life care were considered policy priorities, which included chapters or sections devoted to this topic or explicit mention of priority status.

Palliative and end of life care requires a holistic, individualised approach. Achieving this relies upon effective communication and collaboration with the patient and their carers, and participation from a range of disciplines [30]. With these principles in mind, in the second phase we used thematic analysis [31] to identify health and social care policy recommendations relevant to, but not explicitly focused on, palliative and end of life care. Thematic analysis uses close examination of data to identify common themes, topics and ideas. Our rationale was to identify the policy context within which palliative and end of life care might fit, and which could be used as an opportunity to widen access to palliative and end of life care. For this phase, each document was comprehensively reviewed and examined in depth to inductively identify themes relevant to, but not explicitly focused on, palliative and end of life care. Notes about potential themes were made on the documents electronically. Each time a new potential theme was identified, this was added to the data extraction form to build a picture of the extent of the themes across the dataset as a whole. Themes were finalised through an iterative process of analysis and discussion among team members. Our multi-professional team included clinical expertise in palliative medicine, community nursing and old age psychiatry, and academic expertise in social science, qualitative research and policy, facilitating broad consideration of themes. Initial coding was undertaken by KES and AT, and further refined through discussion with CJE, JEA, RH, ELS and JG.

## Results

A total of 15 policy documents met eligibility. These included general health and social care strategies from Wales (*n* = 1), Scotland (*n* = 2), Northern Ireland (*n* = 1) and England (*n* = 9), and specialty-specific strategies (*n* = 2, Primary Care). The documents comprised 814 pages in total (mean 54, range 12–136), and were published between 2014 and 2019 (Additional file 1 details the included policy documents).

### Objective 1: the extent to which palliative and end of life care is referred to in national health policies

Of the 15 policy documents included, 12 referred to palliative and end of life care. The most frequently used term was ‘end of*’ (end of life, end of their lives), which was used 100 times over 13 documents. Nine documents included a mention of ‘palliat*’ (25 uses), the words ‘die’ and ‘bereave*’ were used eleven times over eight and five documents respectively, and ‘dying’ and ‘hospice’ were used six times, across two and three documents respectively. ‘Death’ was used nine times across four documents. No policy documents used the word ‘terminal’.

None of the documents examined explicitly prioritised palliative and end of life care; where palliative and end of life care was mentioned, this was usually in the context of a general policy priority. For example, several policy documents used end of life care to illustrate the principle of supporting individual preferences. The Government Revised Mandate to NHS England 2018–19 identified the importance of empowering people to ‘*shape and manage their own health and make meaningful choices…as set out in the Government’s response to the end-of-life care choice review*’ (ID4). Where detail on palliative and end of life care was provided, this was often limited to enabling choice of location of death (IDs 3, 5, 10, 12, 11). End of life care was also mentioned in the context of involving patients in advance care planning (IDs 9, 11, 12), early identification of individuals nearing end of life (IDs 4, 9), and providing support for bereaved family members and carers. Staff training, to help identify and support patients approaching the end of life, was mentioned (ID3).

### Objective 2: health policy priorities that could be used as opportunities for widening access to palliative and end of life care

We explored the 15 documents to identify opportunities for widening access to palliative and end of life care. We identified three inter-related themes: integrated care, personalised care, and carer support (Table 1).

**Table 1:** Overview of themes

	**Overview of theme**	**Policy goals for health and social care**	**Mechanisms for delivery**
**Theme 1: Integrated Care**	Re-organisation of services is proposed as a way to improve care delivery; this is proposed through ‘integration’ of existing services. Integrated care is a general term that includes integration of health and social care, of primary and secondary care, of generalist and specialist care, and of physical and mental health care	Present across almost all of the documents: - To ‘dissolve the divide’ between services, to ensure seamless and co-ordinated care for patients (IDs 1, 2, 3, 4, 5, 6, 7, 9, 10, 14, 15) - To reduce avoidable hospital admissions, thereby reducing unnecessary expenditure (IDs 1, 2, 3, 6, 9, 10, 11, 12, 13) - To reduce delayed transfers out of hospital to reduce pressure on the emergency services and the NHS (IDs 1, 2, 3, 10, 11, 12) - To improve access to, and quality of, care in the community to enable people to stay at home and remain independent (IDs 1, 2, 3, 4, 8, 9, 10, 11, 12, 13, 14, 15) - To enable personalised care, with services tailored around the patient to allow multiple needs (e.g. health and social or mental and physical) to be addressed at the same time (IDs 1, 4, 5, 6, 9, 10, 12, 13, 11)	- New models of care: Introduction of Integrated Care Systems and Sustainability and Transformation Partnerships between the NHS and local councils to develop more co-ordinated services and agree on system wide priorities (IDs 2, 3, 4, 6, 7, 15) The Vanguard Programme (IDs 1, 2, 6, 10) was an NHS initiative that focused investment on new models for care delivery, which may be adopted by different CCGs, allowing them to tailor healthcare delivery to suit local pressures. Models included 1) Multispecialty Community Providers (MCP’s); 2) Primary and Acute Care Systems; 3) Acute Care Collaborations (ACCs); 4) Enhanced Health in Care Homes Increased investment in primary care in the community to enable expansion of GP services through increasing access to out-of-hours weekend and evening appointments (IDs 1, 2, 3, 12, 14, 15) Modernised community facilities with MDTs embedded around GPs to increase access to a wider range of specialist resources in the community, and Primary Care Networks to integrate GP practices with community teams (IDs 1, 2, 3, 11, 12, 13, 14, 15) Increased focus on public health and prevention, to minimise the need for hospital admissions (IDs 2, 12, 13, 11) - New funding mechanisms: Integrated personal budgets for both health and social care, to allow for multiple patient needs to be addressed under a single assessment (ID5) - New technology: Digital health that enables people to get the help they need at home, such as remote video consultations and monitoring equipment to avoid unnecessary trips to hospital (IDs 1, 2, 3, 6, 9, 10, 13, 11) Simplified online booking processes and the use of apps to help people manage their own health (IDs 2, 6, 10) Technology infrastructure that improves communication between different systems (IDs 2, 3, 6, 9, 10)
**Theme 2: Personalised Care**	Personalised care allows people to shape and manage their own care according to their preferences, and to make meaningful choices Personalised care is considered a way of improving the efficiency for health and social care by ensuring timely access to services with provision tailored to individual needs. This approach intends to maximise benefit and reduce harm and waste from overtreatment	The goals ranged from aspirational to more specific: - An ambition to provide ‘person-centred care’, with a focus on a holistic approach (IDs 1, 2, 3, 4, 5, 6, 11, 12, 13, 14) - The goal of personalised care is more satisfaction with care, better outcomes, more control, and less tick-box medicine (IDs 5, 6, 15) - Aim to empower patients to take a greater role in their treatment and care, to provide more autonomy (IDs 1, 2, 3, 4, 5, 6, 11, 12, 13, 14) - Personalised care improves the delivery of community care and reduces avoidable hospital admissions (IDs 1, 2, 10, 11) a consequence of this is less medical intervention (ID12) - Personalised care will lead to reduction in inequalities in health care outcomes (IDs 4, 9) -Where specific goals of personalised care were mentioned these were frequently around providing choice over place of care (and place of death) (IDs 1, 2, 3, 11, 13)	- New ways of managing budgets for health and social care including Personal Health Budgets, Integrated Budgets and Integrated personal Commissioning (IDs 1, 2, 3, 4, 5, 6, 10, 13, 15) - Encouraging healthcare professionals to utilise ‘social prescribing’, where patients are referred to a wide range non-clinical local support services, such as befriending schemes or exercise classes, with the aim of addressing patients’ conditions in a more holistic manner (IDs 1, 2, 3) - Use of new technologies and digital tools, such as paperless health records, online appointment scheduling, wearable monitoring technologies and health promoting apps. (IDs 1, 2, 3, 11, 12) -An aspiration to move towards coproduction, ensuring a more equal partnership with patients when making decisions and encouraging discussion between healthcare services and patient. (IDs 1, 2, 4, 5, 6, 7, 11, 12, 13, 14, 15) - Acknowledgement that self-management of health conditions will be aided by increased health literacy, and needs to be underpinned by long term culture change (IDs 2, 3, 4, 7, 11, 12, 13, 14, 15) - Targeting treatments and interventions to a patient’s genetic make-up is an important component of personalised care (IDs 1, 2, 3)
**Theme 3: Support for unpaid carers**	Carers are recognised as a potentially vulnerable group who contribute greatly to the economy. The overriding message is to improve the support provided to carers, both in their caring role and in their lives outside of caring	- To help carers avoid reaching ‘breaking point’ as a result of carer burden, resulting in themselves or the person for whom they care requiring admission to hospital (ID 2) - To reduce the mental and physical health inequalities that carers face (IDs 2, 3, 4, 5, 10, 11, 12, 14) - To support carers’ ability to care, to avoid hospital admission for the carer and dependent (ID2) - To enable carers to continue their critical contribution to society (IDs 13) and sustaining the NHS (ID1) - To empower carers, and increase their involvement in the care of their dependent (ID 2, 4, 12)	- Facilitate access to improved support through better and early identification of carers (IDs 1, 2, 4, 5, 9, 15) - Increased support through financial aid, for example the provision of personal health budgets that encompass carers needs as well as those for whom they care (ID 5, 13) - Initiatives proposed to increase carer independence, such as improving provision of respite care and advocating the right of carers to build careers and meaningful relationships outside of caring responsibilities (ID 13) - Introduction of a ‘carer passport’ to identify carers, allowing healthcare staff to involve them in a patient’s care (IDs 3, 4). This contributes to the wider aim of encouraging shared decision making (IDs 2, 3, 5, 12, 14) and empowering carers to choose the course of care (IDd 4, 11, 13)

Themes identified as opportunities for widening access to palliative and end of life care, the policy goals they fulfill, and mechanisms by which they may be achieved

#### Integrated care

All 15 policy documents included a focus on re-organisation of services and care as a way to enable improvement. This was framed around ‘integration’. For example, the Five Year Forward View advocated ‘*breaking out of the artificial boundaries between health and social care, between generalists and specialists*’ (ID1). This document, and the ‘Next Steps on the NHS Five Year Forward View’ spoke of ‘*triple integration’*; of primary and specialist hospital care, of physical and mental health services, and of health and social care (IDs 1,2).

Mechanisms for integrated care included new models of care, new funding systems and new technologies. New models of care included combining general practice (primary care) and hospital services (ID1), co-ordinating health and social care across communities and integrating general practices with community teams (IDs 1, 2, 3, 4, 6). New funding models were focused around shared budgets, for example “*integrated personal budgets*” that move seamlessly between health and social care (ID5). New technologies included simplified online booking processes, health-promoting apps, and improved information and communication technology such as paperless health records (IDs 1, 2, 3, 6, 9, 10, 11, 13). Where specific groups of beneficiaries were mentioned, these frequently included older people and people with chronic multimorbidities (ID 4,14). The Five Year Framework for GP Contract Reform identified patients with palliative care needs as particularly benefitting from better integrated care (ID15).

#### Personalised care

Personalised care was mentioned in 12 policy documents and conceptualised as allowing people to shape and manage their own health according to their preferences, and to make meaningful choices (IDs 1,2,4,10,11,12,15). It was described as person centred (rather than condition focused), holistic and targeted care (IDs 11,15). The overlap between personalised care and integrated care was clear. For example, improving holistic and personalised care and support planning was considered to help make the NHS more sustainable by reducing avoidable hospital admissions (ID15).

The goal of personalised care was frequently non-specific, such as improved satisfaction, better outcomes, more control, greater autonomy (IDs 5,6,15) and reduction in inequalities (IDs 4,9). Patient choice over where to receive care, improving delivery of community care, and reducing avoidable readmissions was emphasised (IDs 1, 2, 10, 11). One policy stated ‘personalised care might mean less medical intervention’ (ID12).

Mechanisms to achieve personalised care included: (i) new ways of managing budgets for health and social care e.g. (IDs 1,2,3,5,6,10,13,15); (ii) use of social prescribing to address conditions in a more holistic manner (IDs 1,2,3); (iii) use of new technologies and digital tools (IDs 1,2,3,12); (iv) co-production and working in partnership with patients, carers and communities in decision making (IDs 1,2,4,5,6,7,11,12,13,14,15); (v) longer-term societal culture change, including better communication and health literacy (IDs 2,3,4,7,11,12,13,14,15).

#### Support for unpaid carers

The welfare of unpaid carers was cited as a priority in 13 of the documents. Unpaid carers were recognised as a vulnerable group (IDs 1,3,4), particularly those under 18 years, over 85 years, and those with complex and multiple long-term conditions (ID3). The intention was to enable unpaid carers to live a more independent lifestyle, and balance caring with their own needs (ID15). In doing so, hospitalisation for carers and those they care for may be avoided (ID2). There was an emphasis on shared decision making and co-production between staff and carers (IDs 2,3,4,12,13,14).

Mechanisms to improve support for unpaid carers included (i) better identification of carers (IDs 1,2,4,5,9,15); (ii) Personal Health Budgets that allow for carers’ needs as well as the person they are caring for (ID 5,13); (iii) introduction of ‘carer passports’ which would enable health care professionals to identify and recognise carers, involve them in a patient’s care and improve access to information for carers (ID 3,4).

## Discussion

In this documentary analysis we found that most UK health policies studied included some mention of palliative or end of life care; however, this was often to illustrate a general policy priority and details about what should improve, or mechanisms to achieve this, were sparse. Few policy documents referred to a specific palliative or end of life care strategy. Outside of any specific mention of palliative and end of life care, three health policy priority themes were identified that are highly relevant to palliative and end of life care and could be used as opportunities for widening access. These were integrated care, personalised care, and carer support.

Although there is rapidly increasing demand for palliative care [3], we found that the national health policies examined did not include palliative and end of life care as a specific priority. A previous documentary analysis found that palliative and end of life care is rarely prioritised in regional health and wellbeing strategies in England [26]. Similarly, a documentary analysis of international health care policies for older people found inconsistent inclusion of palliative care components [32]. Content relating to palliative and end of life care was brief and often was used to illustrate a more general policy priority. This was frequently the principle of patient choice, with an assumption that patients would choose to die in their usual residence (i.e. at home or in a care home), which may not reflect individual preferences [33].

Good policy has been described as occurring where politics, evidence and delivery align. Low prioritisation of palliative and end of life care in the policies studied could reflect weak political support for palliative and end of life care, and/or lack of awareness, meaning that information on evidence and delivery is not used to improve care. Outside any specific focus on palliative and end of life care (objective 1), we therefore sought to explore policy priorities that may provide an opportunity to improve political support and thus access to palliative and end of life care (objective 2). We identified three inter-related policy priorities that may provide opportunities to improve provision of palliative care. These were integrated care, personalised care, and carer support. While the goals of integrated care were system based with a focus on restructuring service delivery, both personalised care and carer support focused on direct individual benefits. However, there was considerable overlap between the three priorities, seen most clearly through a common goal to reduce acute hospital unplanned admissions and support people to remain in their usual place of care. The service level reform of health and social care service delivery as part of integrated care was portrayed as essential in allowing individuals to shape their own health and care through shared decision-making, key principles of personalised care and carer support. While the goals of these priorities were clear, mechanisms to achieve them were frequently non-specific.

There is strong evidence that palliative care can contribute to meeting the goal of reducing acute unplanned hospital admissions. Indeed, palliative care is one of the few interventions that has been shown to successfully enable individuals to remain in their usual residence, and potentially reduce burdensome transition to acute services [34]. Involvement of specialist palliative care teams improves patient outcomes and patient and caregiver satisfaction [7]. Population-based studies have found specialist palliative care services are associated with improved short-term and long-term carer outcomes [35]. For patients and carers, palliative care delivers personalised care through effective symptom control and skilful communication adapted to individuals, enhancing patients’ sense of security [36]. Palliative care therefore provides an evidence-based approach through which to achieve the three policy priorities. Framing palliative care as a way to deliver these priorities would align evidence and delivery with politics and could lead to improved access.

### Strengths and limitations

Employing a truly systematic strategy for inclusion of policy documents was not possible, since there is no standard policy document repository. Our search strategy was guided by methods for applying systematic search strategies to identify web-based resources in grey literature [27], but remains open to bias. While decisions on which policies to include were based upon clear eligibility criteria, reached with expert input and after discussion among the team, it is possible that some policies which may reasonably have been included were missed. Previous policy analyses have employed similar search strategies, though without the addition of multidisciplinary expert input [37]. We excluded policies specifically related to palliative and end of life care, which by definition would be classed as prioritising palliative and end of life care, and those which were disease or population specific, as we were interested in general health and social care policy. We acknowledge that inclusion of these documents may have identified additional priorities. We limited our study to government policy, and therefore did not include that produced by other organisations such as professional bodies, charities and regulatory organisations. The search terms used to systematically search each document (objective 1) were based on those used in a previous study of regional health policies in England [26]. A newly developed reference framework for identifying inclusion of ten components of palliative care in policy documents provides an opportunity to develop this work in the future [32].

Thematic analysis to identify opportunities for widening access to palliative and end of life care (objective 2) relied on the team’s understanding and experience of the subject area, which may have led to bias in the themes identified. Our two-stage analysis, where first the extent to which palliative and end of life care is mentioned was identified, and second the policy priorities that could be used as opportunities for widening access were identified, can be applied to other policy areas. Our intention was not to critique the strengths and limitations of the policy priorities identified, but to understand how they align with palliative and end of life care.

### Implications for policy, research and practise

National and international calls to strengthen palliative care through policy have had limited success [38]. The brief references to palliative care identified in policies in this study imply that a comprehensive, national level effort to improve palliative and end of life care is not a major priority for policy makers in the UK. Even though palliative care is increasingly recognised as a human right, the role of palliative care can be poorly understood and recognised, which may hinder political support [39]. Indeed, palliative care was notably absent from the WHO guidance on maintaining essential health services during the COVID-19 pandemic, even though it is arguably an essential component of the pandemic response [40]. Framing palliative care as a way to deliver explicit health priorities may be more effective than direct approaches. Furthermore, aligning palliative care with mainstream health and social care priorities could help to reduce known inequalities in access to care such as by age, socioeconomic position and diagnosis [41–43]. We have identified three clear policy priorities that may be opportunities to promote provision of palliative care in the UK; calls to increase provision of palliative care should be framed with these levers in mind. For ongoing research studies, particularly those testing interventions, inclusion of outcomes relating to the priorities identified will help promote impact following completion of studies. While our results are based on UK data, our methods can be replicated in other world regions to identify locally relevant policy levers.

## Conclusions

Our study has shown that palliative and end of life care are rarely explicit policy priorities in UK health and social care policy. However, improving palliative care may provide an evidence-based approach to achieve policy priorities of integrated care, personalised care and support for unpaid carers. Aligning existing evidence of the benefits of palliative care with these policy priorities may be an effective mechanism to both strengthen policy and improve care for people approaching the end of their lives.

## Supplementary Information

**Additional file 1.** Summary of documents included.

## Data Availability

The documents supporting the conclusions of this article are publicly available. A summary of the documents analysed is included in Additional 1.

## Abbreviation

UK: United Kingdom
