# Supplementary material for: Priorities and opportunities for palliative and end of life care in United Kingdom health policies: a national documentary analysis
Source: BMC Palliat Care. 2021 Jul 14;20:108. doi: 10.1186/s12904-021-00802-6 (PMC8279030; doi:10.1186/s12904-021-00802-6)
Supplement: Supplementary file 1 — Additional file 1. Summary of documents included. [file 12904_2021_802_MOESM1_ESM.docx]

**Priorities and opportunities for palliative and end of life care in United Kingdom health policies: a national documentary analysis**

Katherine E Sleeman^1^: Laing Galazka Chair in Palliative Care, NIHR Clinician Scientist and Honorary Consultant in palliative medicine

Anna Timms^1^: Medical Student

Juliet Gillam^1,2^: Research Assistant

Janet E Anderson^3^: Professor of Quality of Care for Older People

Richard Harding^1^: Herbert Dunhill Professor of Palliative Care & Rehabilitation

Elizabeth L Sampson^4^: Professor of Dementia and Palliative Care

Catherine J Evans^1, 5^: HEE/NIHR Senior Clinical Lecturer and Honorary Nurse Consultant in Palliative Care

*^1^ King’s College London, Cicely Saunders Institute, Faculty of Nursing, Midwifery and Palliative Care, Bessemer Road, London, United Kingdom, SE5 9PJ*

*^2^ Florence Nightingale Faculty of Nursing, Midwifery and Palliative Care, James Clerk Maxwell Building, 57 Waterloo Road, London, United Kingdom, SE1 8WA*

*^3^School of Health Sciences, City, University of London, Northampton Square , London EC1V 0HB*

*^4^Marie Curie Palliative Care Research Department, Division of Psychiatry, University College London, London, United Kingdom and Barnet Enfield and Haringey Mental Health Trust Liaison Psychiatry Team, North Middlesex University Hospital, London, United Kingdom*

*^5^Sussex Community NHS Foundation Trust, Brighton, United Kingdom*

Corresponding author: Prof Katherine Sleeman; King’s College London, Cicely Saunders Institute, Bessemer Road, London SE5 9PJ; [katherine.sleeman@kcl.ac.uk](mailto:katherine.sleeman@kcl.ac.uk)

**Additional file 1:** Summary of policy documents included

| **ID** | **Document** | **Publisher** | **Date of Publication** | **Length (No. of pages)** | **Policy Category** | **Aim/summary** |
| --- | --- | --- | --- | --- | --- | --- |
| 1 | Five Year Forward View | NHS England | 2014 | 41 | Nation specific overarching | This document lay out a vision for the future of the NHS. The purpose was to articulate why change is needed, what that change might look like and how it can be achieved. |
| 2 | Next Steps on the NHS Five Year Forward View | NHS England | 2017 | 75 | Nation specific overarching | The NHS Five Year Forward View set out why improvements were needed on the triple aim of better health, better care, and better value. This plan concentrates on what will be achieved over the next two years, and how the Forward View's goals will be implemented. |
| 3 | The NHS Long Term Plan | NHS England | 2019 | 136 | Nation specific overarching | The NHS long term plan sets out the pathway for a new service model fit for the 21st century. The aim is for patients to receive more options, better support and properly joined-up care at the right time in the optimal care setting. |
| 4 | The Government’s revised mandate to NHS England for 2018-19 | Department of Health | 2019 | 27 | Nation specific overarching | The government’s mandate to NHS England sets its objectives and budget, and helps to ensure that the NHS is accountable to Parliament and the public. The aim is to reaffirm the government’s commitment to the NHS, and continues to set objectives and goals to 2020, as well as some specific things that NHS England should be seeking to deliver in the financial year 2018 to 2019. |
| 5 | Extending legal rights to personal health budgets and integrated personal budgets: consultation response | Department of Health | 2019 | 49 | Nation specific overarching | The document seeks views on giving more people the right to have personal health budgets and integrated personal budgets. |
| 6 | 2019-20 Better Care Fund: Policy Framework | Department of Health | 2019 | 12 | Nation specific overarching | Published to direct the implementation of the Better Care Fund (a programme spanning both the NHS and local government which seeks to join-up health and care services, to allow people to manage their own health and wellbeing) in 2019-20 in April 2019. |
| 7 | NHS England and NHS Improvement funding and resource 2019/20: supporting ‘The NHS Long Term Plan’ | NHS England | 2019 | 15 | Nation specific overarching | This document contains information about NHS England and NHS Improvement’s funding in 2019/20. It also sets out NHS England’s and NHS Improvement’s funding and resource for 2019/20 that will support the transition from Next Steps on the NHS Five Year Forward View to The NHS Long Term Plan |
| 8 | Strategic plan for the next four years: Better outcomes by 2020 | Public Health England | 2016 | 24 | Nation specific overarching | This document sets out how PHE intends to achieve its aims over the next 4 years and outlines the key actions for the year ahead. |
| 9 | Universal Personalised Care: Implementing the Comprehensive Model | NHS England | 2019 | 60 | Nation specific overarching | Universal Personalised Care: Implementing the Comprehensive Model states how NHS England will provide Personalised Care for up to 2.5 million people by 2023/24. It is the action plan for the rolling out personalised care across England. |
| 10 | A Healthier Wales: our Plan for Health and Social Care | Welsh Government | 2018 | 38 | Nation specific overarching | This plan aims to improve health and independence of people in Wales. |
| 11 | A National Clinical Strategy for Scotland | Scottish Government | 2016 | 88 | Nation specific overarching | Scotland's National Clinical Strategy sets out ideas on how NHS Scotland needs to change to ensure health and social care services are fit for the future. |
| 12 | Health and Social Care Delivery Plan | Scottish Government | 2016 | 39 | Nation specific overarching | This national delivery plan sets out a series of key actions for government and local health and care services, to deliver better patient care and better population health. |
| 13 | Health and Wellbeing 2026: Delivering Together | Department of Health (NI) | 2016 | 28 | Nation specific overarching | This plan was the response to the report produced by an Expert Panel tasked with considering the best configuration of Health and Social Care Services in Northern Ireland. |
| 14 | The 2018 General Medical Services Contract in Scotland | Scottish Government | 2017 | 74 | Nation specific speciality specific | This contract sets out the distinctive new direction for general practice in Scotland which aims to improve access for patients, address health inequalities and improve population health including mental health, provide financial stability for GPs, and reduce GP workload through the expansion of the primary care multidisciplinary team. |
| 15 | Investment and evolution: A five-year framework for GP contract reform to implement the NHS Long Term Plan | NHS England | 2019 | 108 | Nation specific speciality specific | This briefing summarises the changes set out in the GP contract, and explores the potential implications for trusts and their local partnerships with primary care. |
